# Prospective reporting of statistical analysis plans for randomised controlled trials

**DOI:** 10.1186/s13063-020-04828-8

**Published:** 2020-10-28

**Authors:** Karla Hemming, Anna Kearney, Carrol Gamble, Tianjing Li, Peter Jüni, An-Wen Chan, Matthew R. Sydes

**Affiliations:** 1 Institute of Applied Health Research, University of Birmingham, Birmingham, UK; 2 Biostatistics Department, University of Liverpool, Liverpool, UK; 3 Department of Ophthalmology, School of Medicine University of Colorado, Aurora, USA; 4 Applied Health Research Centre (AHRC), Li Ka Shing Knowledge Institute of St. Michael’s Hospital, Department of Medicine and Institute of Health Policy, Management and Evaluation, University of Toronto, Toronto, Canada; 5 Department of Medicine, Women’s College Research Institute, University of Toronto, Toronto, Canada; 6 MRC Clinical Trials Unit at UCL, Institute of Clinical Trials & Methodology, London, UK

In 2017, JAMA published a statistical analysis plan (SAP) guidance document for randomised clinical trials (RCTs). This guidance is part of the EQUATOR Network of reporting resources and includes a checklist of *minimum* items for reporting details of statistical analysis of RCTs [1]. While the clinical trial protocol should describe the principal features of the statistical analysis, a separate detailed SAP containing sufficient information to support replication by an independent statistician may be needed [2–4].

Given the influence of statistical decisions on trial conclusions, well-documented statistical conduct is essential for transparency and reproducibility of the research. Additionally, pre-specification is important to reduce the occurrence of, and facilitate the detection of, bias particularly in relation to selective analysis and reporting [5, 6]. While guidance exists on the content of protocols [2] and final reports for clinical trials [7], both of which require at a minimum a summary of the statistical analyses, there was until recently no guidance on SAP content. Consequently, there is marked variation in the level of detail provided to allow full replication of statistical analysis. The development of guidance for the content of SAPs aims to reduce this variation and to promote full and transparent reporting of pre-specified analyses [1].

*Trials* therefore encourages the submission for publication of SAPs, which are in line with the guidance document [1], in any of the following formats:

(i) A section directly integrated in the protocol being submitted for publication (for uncomplicated RCTs this might be sufficient);
(ii) An appendix within the protocol being submitted for publication;
(iii) An addendum submitted subsequent to the published protocol paper in *Trials* as a newly peer-reviewed update and which subsequent to publication would be treated much like an appendix to the protocol;
(iv) A separate stand-alone, fully published article, with its own author list and DOI, referencing an already-published protocol (in *Trials* or elsewhere).

These options underscore the importance of the publication of SAPs but also ensure that the reporting of SAPs as separate publications is not made mandatory. For many RCTs, the statistical analysis plan is likely to be uncomplicated and feasibly be defined in full at the time of writing the protocol. For more complex trials, such as those with adaptive designs or those using new methodologies, the development of the SAP might take longer than that of the protocol. *Trials* is actively encouraging researchers to publish their SAPs in the format that fits the complexity of their analysis plan. Whatever option is selected, all authors are encouraged to use the SAP reporting guideline [1]. Both authors and reviewers are therefore tasked with ensuring both reporting according to the SAP reporting guideline [1] as well as the SPIRIT guidance for the protocol as a whole [2].

Different approaches to peer review are anticipated depending on the style of submission and consist of the following:

(i) If the SAP is submitted as part of the protocol, or an appendix to the protocol, peer review of the SAP will be undertaken in conjunction with review of the protocol following current *Trials* guidance for protocols which includes checking against the SAP reporting standard [8].
(ii) If the SAP is submitted as an addendum to an already published protocol in *Trials*, authors will be expected to verify and demonstrate reporting against the SAP reporting guideline and the submission will undergo review as deemed appropriate by the handling editor and which might consist of review by the editor only.
(iii) If the SAP is submitted as a separate publication, the submission will undergo peer review (for those that contain novel or more complicated statistical analysis proposals this would include statistical review) and the SAP would be checked against the SAP reporting standard.

By allowing a number of approaches, *Trials* encourages proportionate review. High-quality peer review has to be balanced against the availability of the academic community to serve as reviewers in times of increasing demands. Thus, peer review might be treated somewhat like a scarce resource. Mandating that every trial must have a published protocol *and* a separate published SAP may well be beyond the timely reviewing capacity of the trial’s community and so should be reserved for those trials for which the level of detail required in the SAP cannot be incorporated into the protocol. Authors should be mindful that these approaches do not distract from relevant legislation and guidelines on when SAPs should be competed.

*Trials* would thus like to encourage the early publication of SAPs and the use of the SAP guidance and checklist for peer reviewing SAPs put forward for publication. Full details on the submission process for the different types of submissions, along with examples, are available [8]. Regardless, we encourage journals to include the protocol and SAP as online addenda to each peer reviewed results paper they publish [9]. SAPs, like protocols, are living documents: SAPs may need to be updated, perhaps in response to new methods or emerging external evidence. In these instances, amendments can be made by (i) submitting an amendment as an update to either the protocol paper or SAP paper, or (ii) documenting changes to the SAP as part of the published final report (likely to be appropriate for minor changes), as per CONSORT guidance for changes which occurred during the trial [10]. Amendments should also be recorded on the relevant clinical trials registration site and with other relevant bodies as appropriate. Authors are requested to ensure clarity when publishing the SAP within a protocol as to whether it the preliminary or final version.

These different approaches to publication not only reflect differences in nature of complexity of SAPs but also promote opportunities for statisticians to take lead authorship positions. The approach endorsed by *Trials* will allow appropriate reflection of the level of academic credit that should be accredited to statisticians for their role in planning of randomised trials. Trials also encourages that funders support requests for publication costs associated with this publication.
